# DIA-based proteomics reveals anti-inflammatory role of DL-3-*n*-butylphthalide in cerebral small vessel disease-induced brain injury in hypertensive rat

**DOI:** 10.1515/tnsci-2025-0381

**Published:** 2025-08-29

**Authors:** Juan Sun, Zhe Su, Yingxiao Ji, Fangming Wang, Jingru Zhao, Jian Zhang, Litao Li

**Affiliations:** Department of Graduate School, HeBei North University, Zhangjiakou, Hebei, P.R. China; Department of Neurology, Hebei General Hospital, Shijiazhuang, Hebei, P.R. China; Hebei Provincial Key Laboratory of Cerebral Networks and Cognitive Disorders, Shijiazhuang, Hebei, P.R. China; Department of Geriatrics, the Second Hospital of Hebei Medical University, Shijiazhuang, Hebei, P.R. China

**Keywords:** cerebral small vessel disease, DL-3-*n*-butylphthalide, data-independent acquisition mass spectrometry, neuroinflammation

## Abstract

**Objectives:**

Excessive neuroinflammatory responses represent a key pathological mechanism in cerebral small vessel disease (CSVD). Dl-3-*n*-butylphthalide (NBP), a compound previously demonstrated to possess anti-inflammatory properties in ischemic stroke, was investigated for its potential therapeutic effects in a rodent model of CSVD. This study aimed to elucidate the neuroprotective mechanisms of NBP in CSVD pathogenesis.

**Methods:**

Forty-week-old spontaneously hypertensive rats were selected as a CSVD rodent model to determine the neuroprotective effects of NBP. Cognitive ability was assessed using the Morris water maze after 28 weeks of treatment. Pathological changes in the brain tissue were observed through immunohistochemistry. Data-independent acquisition (DIA) mass spectrometry was executed to identify the probable targets of NBP in CSVD. Based on the proteomics results, the expression of the toll-like receptor 4 (TLR4)/nuclear factor kappa-B (NF-κB) signaling pathway in the rat hippocampus was evaluated by western blotting and quantitative real-time polymerase chain reaction (qRT-PCR).

**Results:**

NBP treatment ameliorated the cognitive abilities and pathological changes in CSVD. DIA proteomics revealed 262 differentially expressed hippocampal proteins, with bioinformatics analysis highlighting acute inflammatory response as a primary target. Furthermore, western blotting and qRT-PCR results confirmed these results and showed that after treatment with NBP, TLR4 regulated NF-κB pathway and inflammatory factors decreased.

**Conclusions:**

Our findings demonstrated that NBP exerts neuroprotection in CSVD probably by suppressing TLR4/MyD88/NF-κB-mediated neuroinflammation. This study provides the evidence of NBP’s therapeutic mechanisms in CSVD, suggesting its potential as a targeted anti-inflammatory treatment.

## Abbreviations

BBB: blood–brain barrier
CNS: central nervous system
CSVD: cerebral small vascular disease
DEPs: differentially expressed proteins
DIA: data-independent acquisition
GO: gene ontology
HLB: hydrophilic-lipophilic balance
IHC: immunohistochemistry
IKK: inhibitor of kappa B kinase
IL-1β: interleukin-1β
LFB: Luxol fast blue
MWM test: Morris water maze test
MyD88: myeloid differentiation factor 88
NBP: Dl-3-*n*-butylphthalide
NF-κB: nuclear factor kappa-B
qRT-PCR: quantitative reverse transcription PCR
ROI: region of interest
SBP: systolic blood pressure
SHR: spontaneously hypertensive rats
TLR4: toll-like receptor 4
TNF-α: tumor necrosis factor-α
WKY rats: Wistar-Kyoto rats
XIC: extracted ion chromatogram

## 1 Introduction

Cerebral small vessel disease (CSVD) is a neurological disorder caused by structural and functional abnormalities in the microvasculature of brain, including cerebral arterioles, capillaries, and venules [1]. It is characterized by distinct neuroimaging findings such as white matter hyperintensities, cerebral microbleeds, lacunar infarcts, enlarged perivascular spaces, and cortical or subcortical atrophy. These pathological changes contribute to a range of clinical consequences, including ischemic or hemorrhagic stroke, progressive cognitive decline, mood disturbances, and gait abnormalities [2,3]. Evidence indicated that CSVD is mainly attributed to aging and vascular risk factors, including hypertension, diabetes, vasculitis, and amyloidosis. Among these, hypertension is the most important risk factor for CSVD [4]. Long-term hypertension directly causes vascular wall remodeling to adapt to increased pressure, including smooth muscle cell proliferation, collagen deposition, and pathological remodeling of the extracellular matrix, resulting in vascular thickening, luminal stenosis, blood–brain barrier (BBB) destruction, inflammation, and dysfunction of the brain [5–7].

CSVD is thought to be closely related to immune inflammatory mechanisms [8,9]. Microglia and astrocytes are immune cells widely distributed in the central nervous system (CNS) and can be activated by prolonged hypertension, further releasing tumor necrosis factor-α (TNF-α), interleukin-1β (IL-1β), matrix metalloproteinases, and other inflammatory factors that jointly accelerate the inflammatory cascades [10]. Toll-like receptor 4 (TLR4)/nuclear factor kappa-B (NF-κB) signaling, a classic inflammatory regulatory pathway, plays an important role in the pathogenesis of brain injury [11–14]. When the pattern recognition receptor (PRR) TLR4 is activated, it can transmit signals through its key adaptor protein, myeloid differentiation factor 88 (MyD88), to activate the transcription factor NF-κB. Phosphorylated NF-κB promotes the production of inflammatory cytokines and aggravates brain injury [15]. Studies have shown that inhibition of the TLR4/NF-κB pathway can reduce neuroinflammation in rats with cerebral injury [16,17].

Dl-3-*n*-butylphthalide (NBP) is a compound originally extracted from the seeds of A*pium graveolens* Linn. Numerous clinical trials and meta-analyses have shown that NBP can significantly improve functional outcomes in patients with stroke [18]. The possible mechanisms include inhibiting of platelet aggregation, protection of mitochondrial function, reduction of oxidative damage, and suppression of inflammation [19,20]. NBP can inhibit the TLR4/NF-κB signaling pathway in the CNS of mice with spinal cord injury and restrain microglial proliferation and production of pro-inflammatory mediators [21]. NBP may be a promising candidate for the prevention and treatment of vascular dementia because of its antioxidant and anti-inflammatory properties [22,23].

Despite the established efficacy of NBP in acute stroke management, its role in chronic CSVD, particularly in hypertensive contexts, has not been fully characterized. Combining behavioral, histopathological, and data-independent acquisition (DIA)-based proteomic approaches, this study reveals neuroprotective mechanism of NBP through probably suppressing TLR4/NF-κB mediated neuroinflammation in CSVD pathogenesis.

## 2 Materials and methods

### 2.1 Experimental animals and drug administration

Male spontaneously hypertensive rats (SHRs) and Wistar-Kyoto rats (WKY rats) at 12 weeks of age were purchased from the Vital River Laboratory Animal Technology Co. Ltd, Beijing, China. The protocol was approved by the Institutional Animal Care and Use Committee and the local experimental ethics committee. All rats were allowed free access to food and water under controlled conditions (12/12 h light/dark cycle with humidity of 60% ± 5%, 22 ± 3°C).

**Figure 1:**
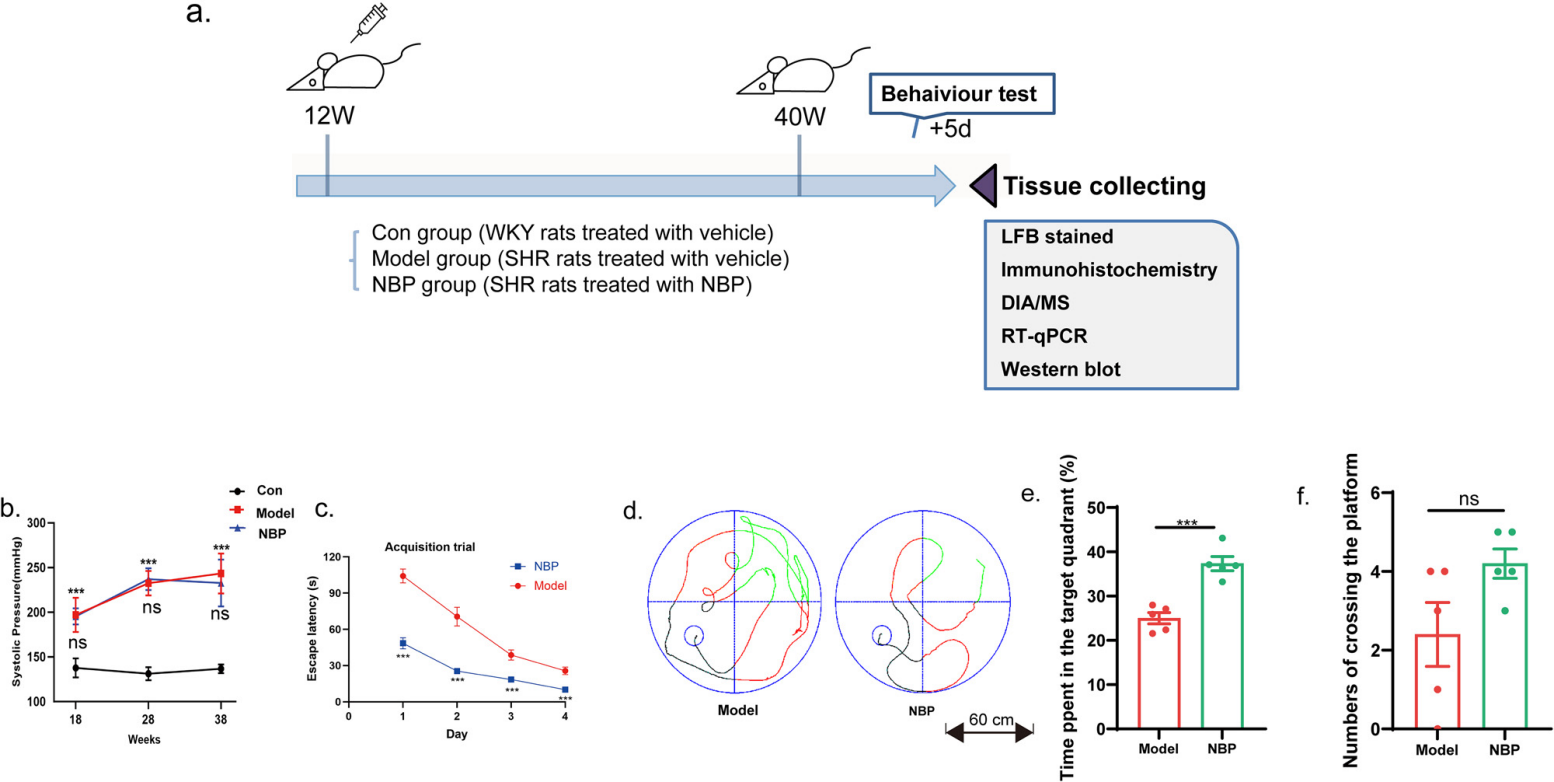
NBP improved cognitive function of SHR rats. (a) Experimental scheme, treatments, and techniques used to evaluate the efficacy of NBP in CSVD rat model. (b) SBP of rats in each group at different time points. ***p < 0.001 compared with the Con group; ns denotes no significant significance between the NBP group and model group. *p* Values from one-way ANOVA with Tukey’s *post hoc* test (*n* = 5). (c) Escape latency to reach the hidden platform during the acquisition trial in the MWM test. *p-*Values from two-way repeated measures ANOVA with Tukey’s *post hoc* test (*n* = 5). (d) Images of swimming path in acquisition trial. (e) Percent time of the target quardrant in the probe trail of MWM test. *p*-Values from one-way ANOVA with Tukey’s *post hoc* test (*n* = 5). (f) The numbers of crossing the platform in the probe trail of MWM test. *p*-Values from one-way ANOVA with Tukey’s *post hoc* test (*n* = 5). *p < 0.05, **p < 0.01, ***p < 0.001, ns denotes no significant significance, compared with the model group.

NBP (purity > 99.5%) was provided by Shijiazhuang Pharmaceutical, Co., Ltd, China and dissolved in corn oil solution. Eighteen male SHRs were randomly divided into two groups: model and NBP (*n* = 9). Six WKY rats of the same age were included in the control group. The rats in the NBP group were intragastrically administered 60 mg/kg/d NBP dissolved in corn oil once a day from 12 weeks of age until 40 weeks, while the control and model groups were administered an equal volume of corn oil. Systolic blood pressure (SBP) was monitored using a noninvasive sphygmomanometer following a previous procedure [24]. After 28 weeks of treatment, the cognitive ability of rats was assessed using the Morris water maze test (MWM test) before being sacrificed. A schematic of the experimental design is shown in Figure 1.

### 2.2 MWM test

The MWM test was conducted to evaluate the learning and memory functions of the CSVD rats (*n* = 5 per group). The MWM test was carried out in a round tank (120 cm in diameter and 60 cm in depth) containing opaque water maintained at 25 ± 2°C, and included a training phase to acquire a hidden platform and a free exploration trial phase. During the 4-day training trials, the average escape delay was recorded. After the final training experiment, the percentage of time spent in the target quadrant during the probe trial was recorded.

### 2.3 Luxol fast blue (LFB) staining

White matter lesions were evaluated by LFB staining. Coronal sections with a thickness of 5 μm were stained with LFB at room temperature in the dark for 12 h. Then the slices were soaked in a 0.05% lithium carbonate solution for 10 s, then in 70% ethanol, and rinsed in ddH_2_O until the gray and white matter could be clearly distinguished. After drying at room temperature, slices were photographed under a light microscope (OLYMPUS, Japan).

### 2.4 Immunohistochemistry (IHC)

Brain sections (5 µm thick) were blocked with 3% H_2_O_2_ and 3% normal goat serum, then incubated, respectively, with the primary antibody: anti-AQP4 (Abcam, ab259318; 1:2,000), anti-NeuN (Sigma-Aldrich, ABN78, 1:100), anti-GFAP (Millipore, MAB360, 1:500), and anti-Iba-1 (Wako Chemicals, 019-19741, 1:250) overnight. The secondary antibody, biotinylated conjugate, and diaminobenzidine were obtained from the SP Rabbit/Mouse HRP Kit (DAB) (Shenzhen Tongying Biotechnology Company, China). Finally, stained brain sections were observed under a microscope. Image analysis was performed blind to treatment groups. All slides were coded by an independent researcher prior to quantification, and decoding occurred only after statistical analysis. Three fields per section were randomly selected from the hippocampus at 400× magnification, avoiding artifacts. Regions of interest were outlined using ImageJ1.52v based on Neun, Iba1, or GFAP staining (*n* = 3 per group).

### 2.5 Total protein extraction, peptide desalting, and quantification

Hippocampal samples (*n* = 3 in the model and NBP groups, respectively) were suspended in protein lysis buffer (8 M urea, appropriate protease inhibitor) and treated with a high-flux tissue grinding machine three times for 40 s each. After centrifugation at 16,000*g* for 30 min at 4°C, the collected supernatant was quantified. Around 100 µg of protein was resuspended in triethylammonium bicarbonate buffer (TEAB) at a final concentration of 100 mM. The mixture was diluted with tris phosphine to a final concentration of 10 mM at 37°C for 60 min and then alkylated with iodoacetamide (final concentration of 40 mM) at room temperature for 40 min in darkness. After centrifugation at 10,000*g* for 20 min at 4°C, the pellet was resuspended in 100 µL TEAB at a final concentration of 100 mM. Then trypsin was added at 1:50 and incubated at 37°C overnight. The enzymatically drained peptides were re-solubilized with 0.1% trifluoroacetic acid, and the peptides were desalted with hydrophilic-lipophilic balance, and drained a vacuum concentrator. Finally, the peptides were quantified using the Thermo Fisher Scientific Peptide Quantification Kit (Item #23275).

### 2.6 DIA mass detection and protein identification

Based on the peptide quantification results, the peptides were analyzed using a Vanquish Neo coupled with an Orbitrap Astral mass spectrometer (Thermo Fisher Scientific, USA) in DIA mode at Majorbio Bio-Pharm Technology Co. Ltd (Shanghai, China). Briefly, an ES906 column (150 µm × 15 cm, Thermo, USA) was used with solvent A (water containing 2% ACN and 0.1% formic acid) and solvent B (water containing 80% ACN and 0.1% formic acid). The peptides were eluted using a 180 SPD gradient at a flow rate of 500 nL/min. DIA-MS data were collected over an *m*/*z* range of 100–1,700. Spectronaut software (version 18) was used to search the DIA raw data. Three peptides per protein and three daughter ions per peptide were selected for quantitative analysis. The parameters were as follows: protein false discovery rate (FDR) ≤0.01, peptide FDR ≤0.01, peptide confidence ≥99%, and extracted ion chromatogram width ≤75 ppm. Shared and modified peptides were excluded, and peak areas were calculated and summed to obtain quantitative results.

### 2.7 Bioinformatics analysis of differentially expressed proteins (DEPs)

*p*-Values and fold changes (FC) for proteins between the two groups were calculated using the R package “*t*-test.” The thresholds of FC (>1.2 or <0.83) and *p*-value <0.05 were used to identify DEPs. Functional enrichment of DEPs was performed using gene ontology (GO) analysis (http://geneontology.org/).

### 2.8 Western blotting

Isolated hippocampal tissue was lysed in pre-cooled RIPA lysis buffer and the supernatant was collected (*n* = 3 per group). The protein content of the extract was determined using the bicinchoninic acid method, followed by electrophoresis and membrane transfer. The membranes were incubated overnight at 4°C with the primary antibodies: anti-TLR4 (Proteintech, 19811-1-AP, 1:800), anti-MyD88 (BOSTER, PB9148, 1:300), anti-p-p65-NF-κB (Cell Signaling Technology, 3033S, 1:500), and β-actin (Bioworld Technology, BS6007M, 1:10,000). The next day, the membrane was incubated with a secondary antibody (goat anti-rabbit/mouse IgG, 1:800) at room temperature for 2 h. Proteins were visualized using an enhanced chemiluminescence kit (Thermo Fisher Scientific, USA) and an imaging system.

### 2.9 Quantitative real-time polymerase chain reaction (qRT-PCR)

Total RNA was extracted from the hippocampal tissue using TRIzol reagent (Invitrogen, Carlsbad, CA, USA), as recommended by the manufacturer (*n* = 3 per group). Reverse transcription was performed using a first-strand cDNA synthesis kit (Fermentas International, Inc., Burlington, Canada). The obtained cDNA was amplified using an qRT-PCR system (Agilent, Palo Alto, CA, USA) in the presence of the corresponding primer and fluorescent dye (SYBR Green I; Cwbio). After normalization to GAPDH, the relative abundance of mRNA was calculated using 2^−ΔΔCt^ method. The forward and reverse primers that were used are listed in Table 1.

**Table 1:** Primers for RT-qPCR

Gene	Forward (5′–3′)	Reverse (5′–3′)
TLR4	CTTTCAGGGAATTAGGCTCC	CCAAGATCAACCGATGGAC
iNOS	ACACAGTGTCGCTGGTTTGA	TCTCCGTGGGGCTTGTAGTT
IL-1β	GGATGATGACGACCTGC	CTTGTTGGCTTATGTTCTG
IL-6	CGTCAGCCGATTTGCCATTT	ACTCAGGCATCGACATTCCG
GAPDH	CCATGGAGAAGGCTGGG	CAAAGTTGTCATGGATGACC

### 2.10 Statistical analysis

Statistical analyses were performed using SPSS 27.0 statistics software. The differences between treatments in escape latency in the MWM task were analyzed using a repeated-measures test, and differences with *p* < 0.05 were considered statistically significant. For DEPs analysis, Welch’s *t*-test was executed for comparisons between the two groups. The thresholds of FC (>1.2 or < 0.83) and *p*-value <0.05 were used to identify DEPs. In GO analysis, multiple testing correction was performed using the Benjamini-Hochberg (BH) method to control the FDR, with significance defined as FDR-adjusted *p* < 0.05. The results of IHC, western blotting, and qRT-PCR were analyzed using a one-way analysis ANOVA, *p* < 0.05. Data were tested for normality (Shapiro–Wilk test) and homogeneity of variance (Levene’s test). Non-parametric tests (Mann–Whitney *U*) were used when assumptions were violated. Data were expressed as mean ± S.E.M.


**Ethical approval:** The research related to animals’ use has been complied with all the relevant national regulations and institutional policies for the care and use of animals. All experiments were approved by the Animal Ethics Committee of Hebei General Hospital (ethical approval number: 2024 Research Ethics Review (071)) and conducted in accordance with the Regulations on the Management of Experimental Animals of Hebei Province.

## 3 Results

### 3.1 NBP improved cognitive function of CSVD rats

Blood pressure was measured in each group every 2 weeks. SBP was significantly higher in both the model and NBP group than that in the control ones from 18th week to 38th week (*p* < 0.001). There was no significant difference between the model and NBP groups, indicating that NBP had no effect on blood pressure (Figure 1b). The SBP results for the rats in each group are presented in Table 2.

**Table 2:** Systolic blood pressure (SBP) of the rats in each group

Group	SBP (mmHg)		
	18 W	28 W	38 W
Con	137.733 ± 2.758	131.200 ± 1.908	136.800 ± 1.281
Model	197.000 ± 4.956***	232.533 ± 3.548***	243.333 ± 5.804***
NBP	195.333 ± 2.329***^/ns^	237.067 ± 3.132***^/ns^	232.667 ± 6.780***^/ns^

*p < 0.05, **p < 0.01, and ***p < 0.001 *vs* Con group at the same time point. ns *vs* Model group at the same time point.

In the acquisition trial, significant differences in escape latency were found between the model rats and NBP-treated rats in all four trials (*p* < 0.001, Figure 1c). Furthermore, in the probe trial, when the platform was removed, the percent of time spent in the target quadrant in the NBP-treated group improved significantly (*p* < 0.001, Figure 1e). During the free-swimming phase, although no statistically significant difference was observed in platform crossings between the two groups, the NBP group exhibited a higher number of crossings compared to the model group (4.2 ± 0.374 in the NBP group versus 2.4 ± 0.812 in the model group, *p* > 0.05, Figure 1f). This evidence indicated that the spatial memory of the rats in the NBP group was improved compared to that of vehicle-treated rats.

### 3.2 NBP relieved CSVD-induced demyelination in the corpus callosum, and loss of neurons, activation of microglias and astrocytes in the hippocampus

**Figure 2:**
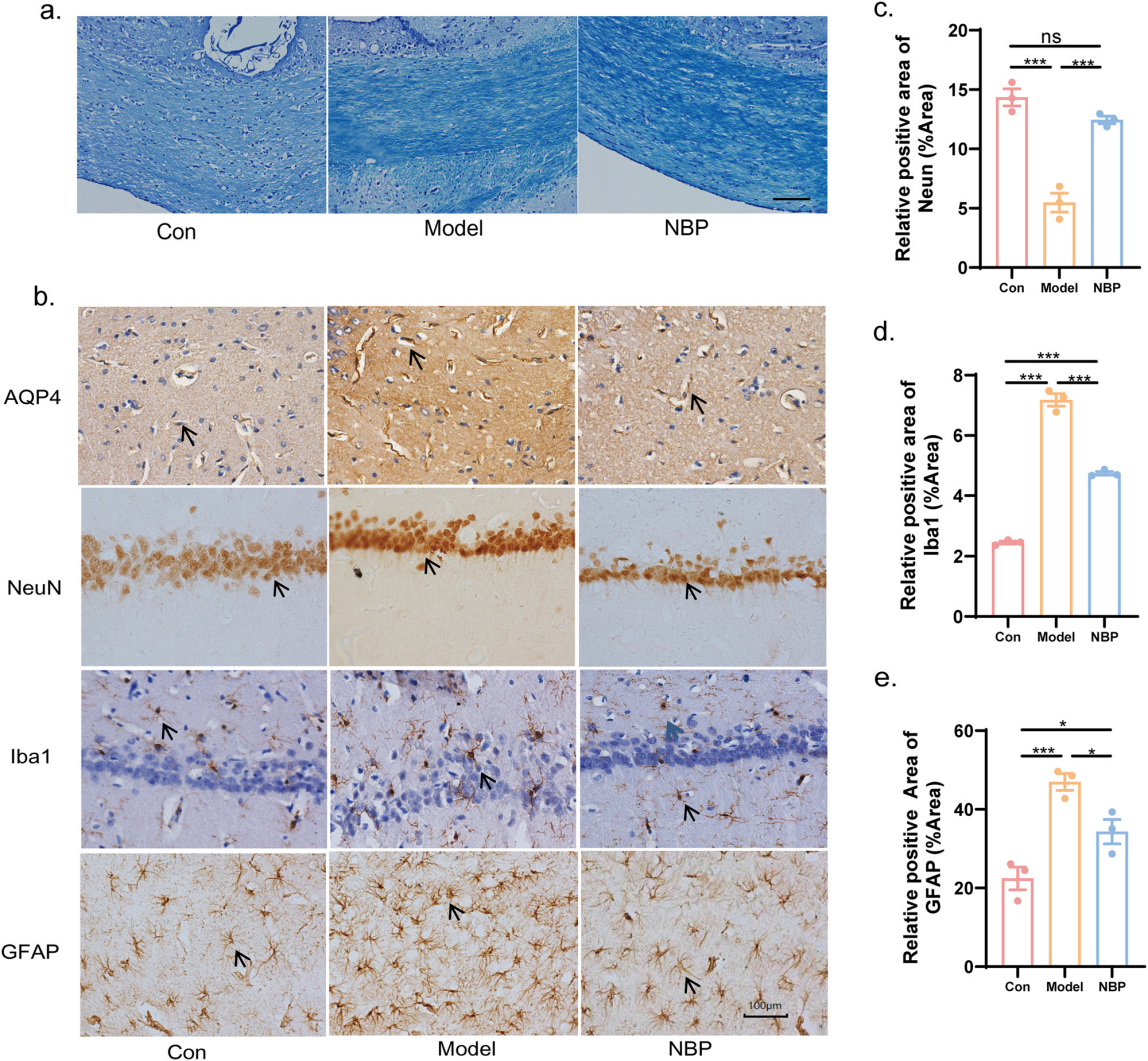
NBP relieved CSVD-induced demyelination in the corpus callosum, and loss of neurons, activation of microglias and astrocytes in the hippocampus. (a) Representative recordings of LFB staining in the corpus callosum of CSVD rats. Scale bar = 200 µm. (b) Immunohistochemical staining of AQP4, NeuN, Iba1, and GFAP in the hippocampus of CSVD rats at 40 weeks (400× magnification). Scale bar = 100 µm. Histograms of IHC illustrating the expression of NeuN (c) was significantly increased in NBP group compared with the model one, while Iba1 (d) and GFAP (e) was decreased in the NBP group. *p*-Values from one-way ANOVA with Tukey’s *post hoc* test (*n* = 3). *p < 0.05, **p < 0.01, ***p < 0.001, ns denotes no significant significance, compared with the model group. *p < 0.05, **p < 0.01, and ***p < 0.001.

The myelin sheath was identified by LFB staining. The results showed a decrease in the optical density and disordered arrangement of myelin in the corpus callosum of rats in the SHR model group, indicating hypertension-induced demyelination. However, treatment with NBP significantly increased the optical density of LFB myelin staining in SHRs (Figure 2a).

Elevation of Aquaporin 4 (AQP4) is an important factor in BBB breakdown. The results showed an increase in AQP4^+^ in the hippocampus of SHR rats, whereas the administration of NBP decreased the expression of AQP4^+^ in the SHR model group rats, indicating alleviation of BBB breakdown (Figure 2b).

IHC staining for NeuN was performed to observe the loss of neurons in the hippocampus of SHR. As shown in Figure 2b and c, long-term hypertension induced a significant loss of hippocampal neurons, which was relieved by treatment with NBP. The relative positive area of NeuN^+^ (%) was 5.472 ± 0.801 in the model group versus 14.345 ± 0.714 in the control group (*p* < 0.001) and 12.441 ± 0.316 in the NBP group versus 5.472 ± 0.801 in the model group (*p* < 0.001). We also performed IHC staining for Iba1 and GFAP in each group to reflect the activation of microglia and astrocytes. Hypertension-induced CSVD triggered microglial and astrocyte activation in the hippocampus (*p* < 0.001 for both), which was inhibited by NBP (Figure 2b, d, and e). The relative positive area of Iba1^+^ (%) was 4.744 ± 0.062 in the NBP group versus 7.178 ± 0.211 in the model group (*p* < 0.001). The relative positive area of GFAP^+^ (%) was 34.333 ± 3.091 in the NBP group versus 46.975 ± 2.132 in the model group (*p* < 0.05).

### 3.3 Quantitative proteomic analysis of hippocampus in the NBP-treated CSVD rats

**Figure 3:**
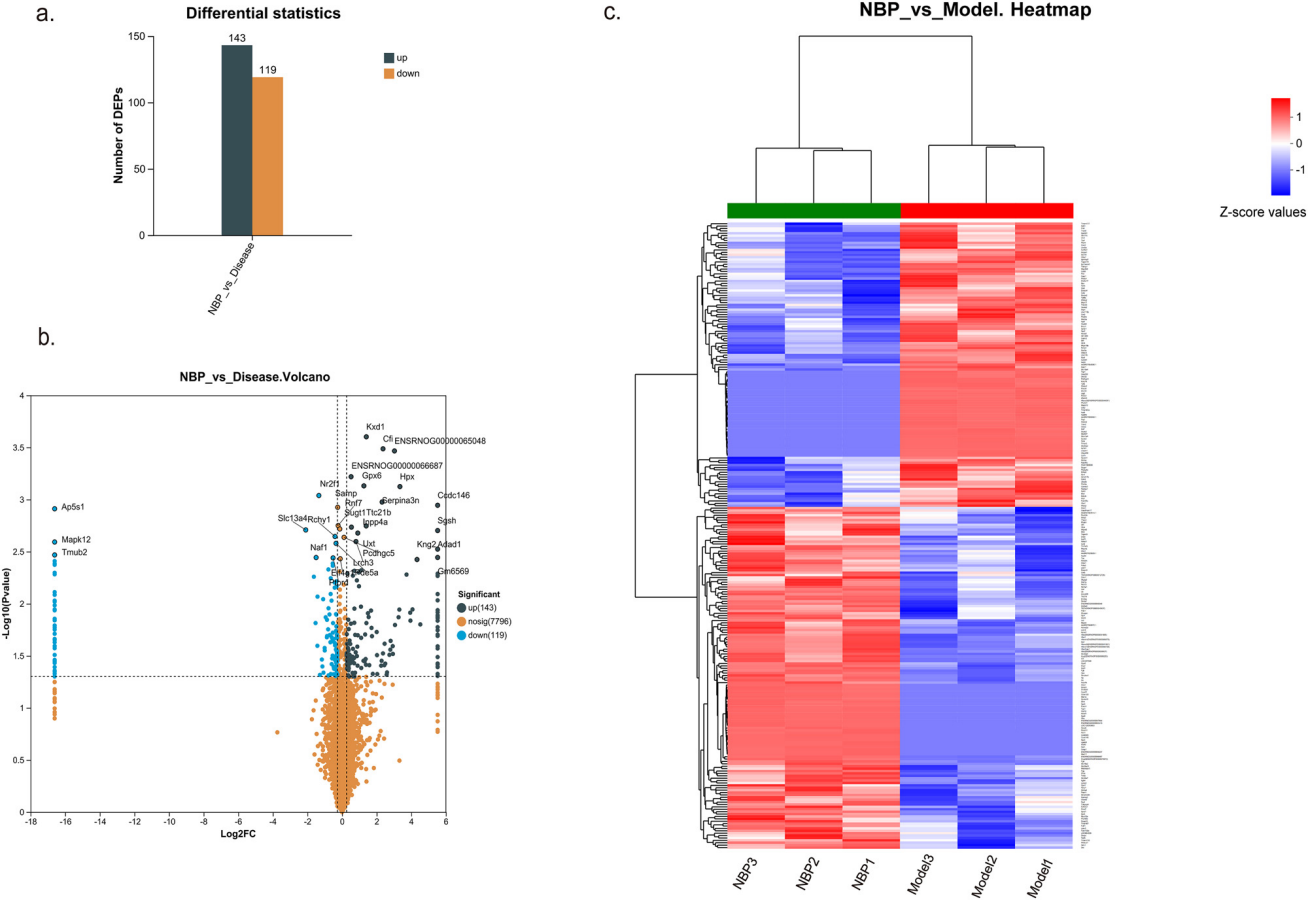
Quantitative proteomic profile of hippocampus in the NBP-treated CSVD rats and none treated ones. (a) The histogram shows 143 up-regulated and 119 down-regulated hippocampus DEPs in NBP group compared with the model group. (b) Volcano plot of DEPs of SHR rats in NBP group versus model group. (c) Heatmap of DEP ratios, wherein each column in the figure represents a sample, and each row represents a protein. The color gradient indicates the relative expression levels of proteins across sample groups, with exact *Z*-score values referenced from the color bar scale. The left dendrogram indicates hierarchical clustering of proteins. The closer the two protein branches are, the more similar their expression profiles.Top dendrogram indicates hierarchical clustering of samples. The closer the two sample branches are, the more similar their global protein expression patterns. Sample names are labeled below the heatmap. DEPs were calculated through Welch’s *t*-test between the two groups. The thresholds of FC (>1.2 or <0.83) and *p*-value <0.05 were used to identify DEPs.

To explore the protein changes in the hippocampus of NBP-treated CSVD rats, quantitative proteomic analysis was performed together with DIA mass spectrometry. Using FC (>1.2 or <0.83) and *p*-value <0.05 as the cutoff, 262 proteins were determined as DEPs. Among the 262 DEPs, 143 were upregulated and 119 were downregulated in NBP treatment group compared to the model group (Figure 3a). The complete list of DEPs is revealed in the supporting info (Table S1). Volcano plot showed DEPs between model group and NBP treatment group (Figure 3b). In addition, the DEPs were also visualized by a heatmap in Figure 3c.

### 3.4 GO analysis of DEPs of hippocampus in the NBP-treated CSVD rats

**Figure 4:**
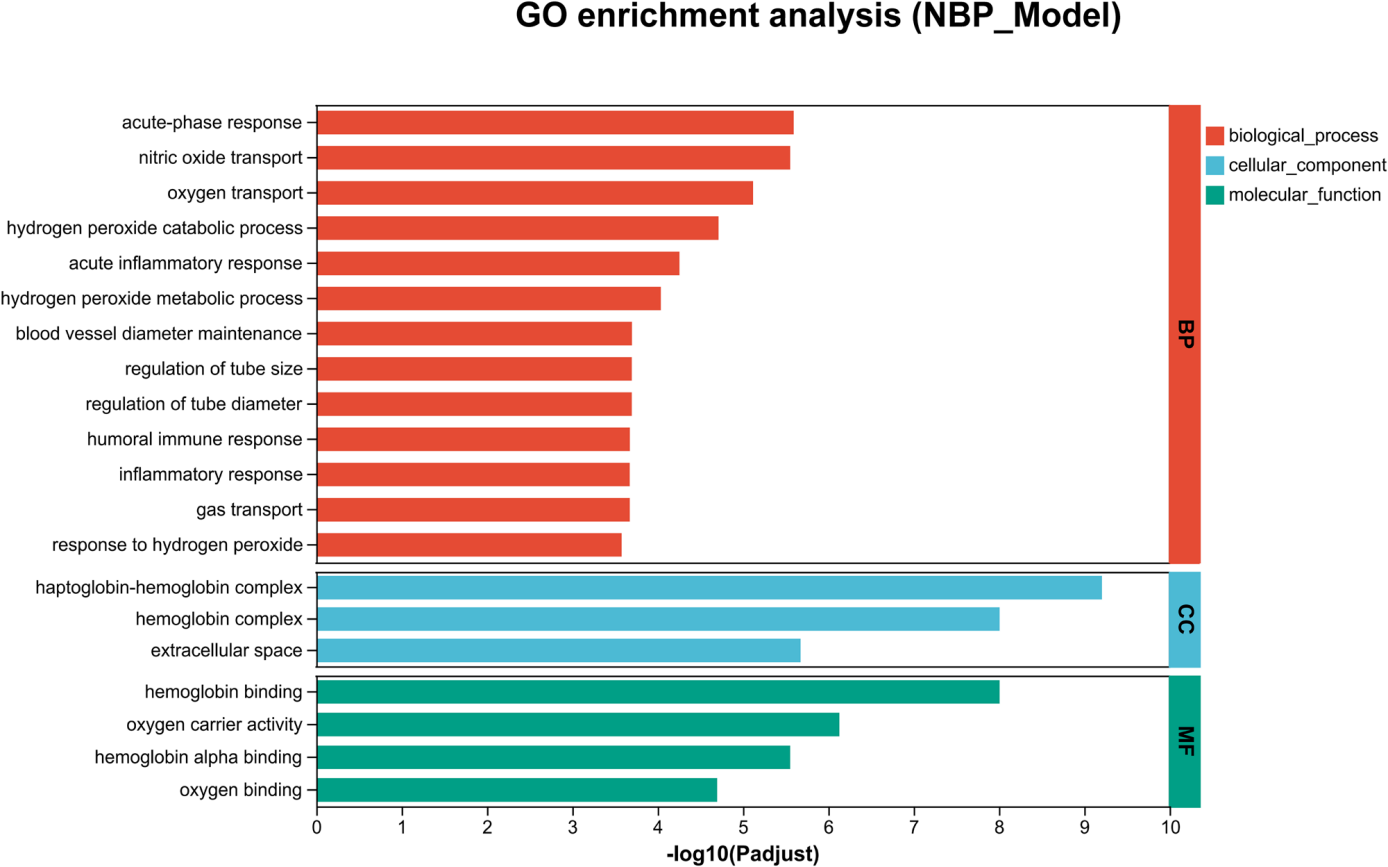
GO analysis of DEPs in NBP- and vehicle-treated SHR rats. Classifcation of the DEPs based on biological process (BP), cellular component (CC), and molecular function (MF). The vertical axis represents the significantly enriched functional classification and pathways, and the horizontal axis represents −log10 of FDR-adjusted *p*-values of enriched result in each classification. FDR-adjusted *p*-values were performed using the BH method to control the FDR.

Functional classification and enrichment analysis of DEPs were performed using GO analysis. The results based on biological processes showed that the significantly altered proteins were involved in the regulation of acute-phase response (FDR*-adjusted p* = 2.556399507 × 10^−6^, Rich factor: 0.43478260869565), acute inflammatory response (FDR-*adjusted p* = 5.5621122703 × 10^−5^, Rich factor: 0.3125), regulation of immune system process (FDR-*adjusted p* = 0.000327606839389, Rich factor: 0.076171875) (Figure 4).

### 3.5 NBP downregulated the expression of the TLR4/MyD88/NF-κB and inflammatory cytokines in the hippocampus of CSVD rats

**Figure 5:**
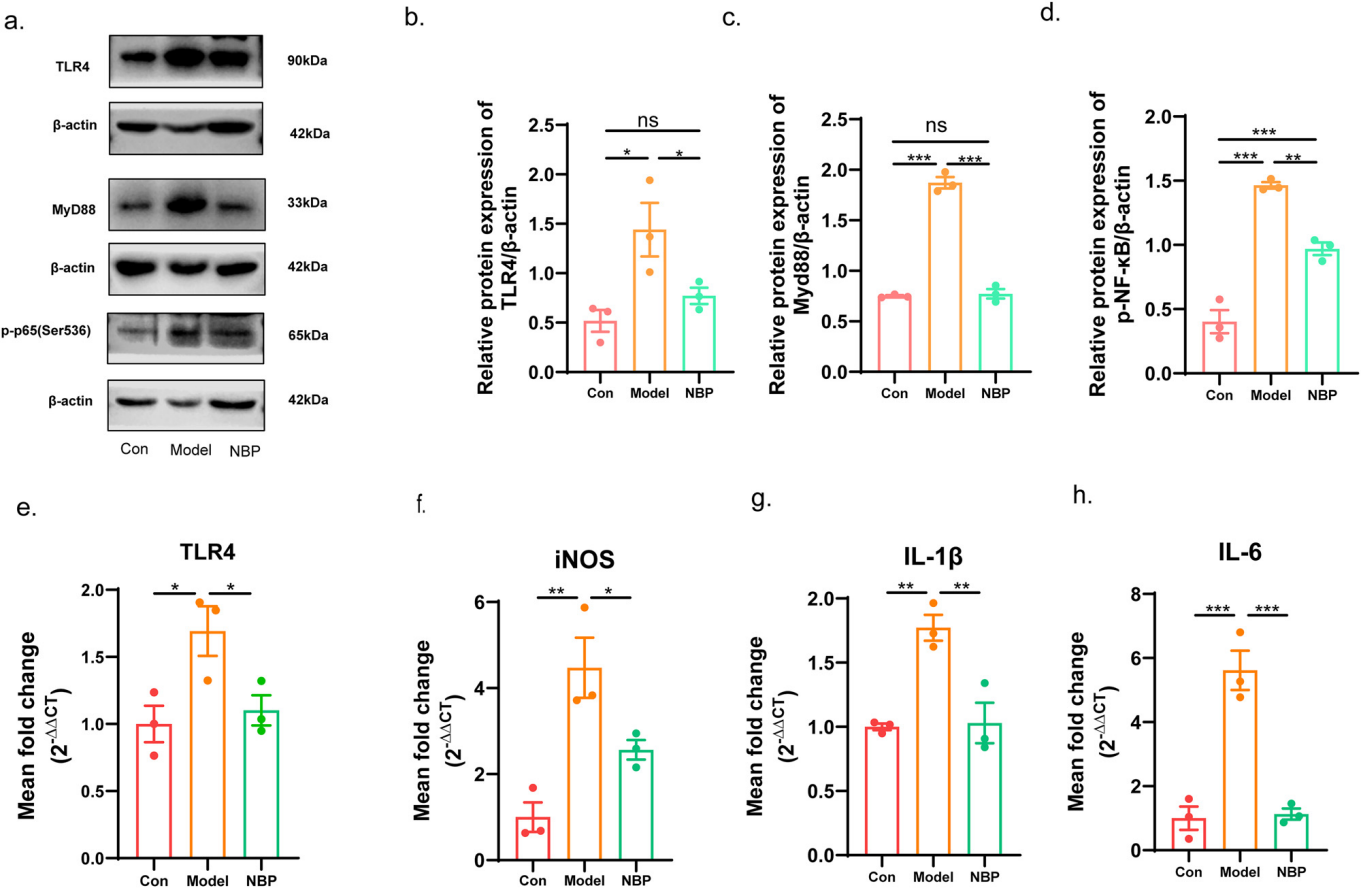
NBP attenuated the expression of the TLR4/MyD88/NF-κB and inflammatory cytokines in the hippocampus of CSVD rats. (a) Representative western blotting of DEPs TLR4, MyD88, and p-p65-NF-κB in each group. The relative level of TLR4 (b), MyD88 (c), and p-p65-NF-κB (d) was quantified. Relative mRNA expression of the TLR4 (e), iNOS (f), IL-1β (g), and IL-6 (h). *p*-Values from one-way ANOVA with Tukey’s *post hoc* test (*n* = 3). *p* < 0.001 is marked as ***, *p* < 0.01 is marked as **, and *p* < 0.05 is marked as *. ns means no significant.

The TLR4-mediated NF-κB pathway serves as a critical regulator of inflammatory responses, and has been extensively studied in cerebrovascular diseases and neurodegenerative diseases [11]. Based on the proteomic results, western blotting was used to detect the protein levels of TLR4/NF-κB in each group (Figure 5a–d). We found that the relative expression of TLR4, MyD88, and p-p65-NF-κB at the protein level was up-regulated in SHR rats (TLR4: *p* < 0.05; MyD88: *p* < 0.001; p-p65-NF-κB: *p* < 0.001). NBP treatment attenuated activation of the TLR4/MyD88/NF-κB pathway (TLR4: *p* < 0.05; MyD88: *p* < 0.001; p-p65-NF-κB: *p* < 0.01).

In addition, we measured the gene expression of TLR4 and pro-inflammatory mediators by qRT-PCR (Figure 5e–h). The relative mRNA expression levels of TLR4, iNOS, IL-1β, and IL-6 were upregulated in CSVD model rats compared with those in control rats, respectively (TLR4: *p* < 0.05; iNOS: *p* < 0.01; IL-1β: *p* < 0.01; IL-6: *p* < 0.001). NBP treatment significantly reduced the expression of TLR4, iNOS, IL-1β, IL-6, and in the NBP-treated CSVD rats compared to that in the vehicle-treated model group (TLR4: *p* < 0.05; iNOS: *p* < 0.05; IL-1β: *p* < 0.01; IL-6: *p* < 0.001).

## 4 Discussion

In this study, we used SHRs as the experimental model, with age-matched WKY rats serving as controls. The MWM test demonstrated that 28-week NBP treatment improved cognitive function in SHRs, accompanied by attenuated glial cell proliferation and activation in the hippocampal region, as well as rescued neuronal degeneration and loss. Further mechanistic investigations revealed a significant downregulation of the TLR4-regulated NF-κB pathway, along with reduced levels of inflammatory cytokines. These findings suggested that NBP exerts neuroprotective effects, likely by suppressing TLR4/NF-κB-mediated neuroinflammation in the pathogenesis of CSVD.

Hypertension is considered to be the main cause of CSVD, and other vascular disease risk factors, including dyslipidemia, diabetes, smoking, and a stressful lifestyle, are also associated with cerebral small vessel lesions [25]. In this study, SHRs were selected as subjects with CSVD to study brain injury induced by long-term hypertension. SHR, rats with primary spontaneous hypertension, are often used as rodent models to study hypertensive-related complications. The spontaneous hypertension rate of SHR is 100% without any interventions [26]. In this study, it was found that the blood pressure of SHR rats was significantly higher than that of control ones. It has been reported that with aging, SHRs develop BBB leakage, astrogliosis, microglial activation, neuronal loss, white matter damage, and brain atrophy, finally leading to declines in learning, memory, and cognitive function. These changes are similar to human CSVD and likely result from chronic hypertension-induced damage to cognition-related brain regions, including the hippocampus [27,28]. Hippocampus, a brain region closely related to mental and cognitive functions, is susceptible to hypoperfusion and hypoxia [29]. Attention to the evaluation of structural and functional changes in the hippocampus will contribute to a deeper understanding of the pathogenesis of CSVD and provide more effective treatment strategies [30]. Our results of LFB and IHC staining showed white matter damage, destruction of the BBB, and proliferation and activation of gliocytes in the CSVD model, which was consistent with previous research [31]. Therefore, SHR can be used as a CSVD rodent model.

NBP has many biological effects and is widely used in the treatment of acute ischemic stroke [22,23]. Currently, we found that NBP improves cognitive function in rats with CSVD in the MWM tests, alleviates pathological changes in the hippocampus, and inhibits the activation and proliferation of microglia and astrocytes. In this study, MWM experiments were designed to assess whether NBP treatment could ameliorate cognitive deficits in SHRs. While our current conclusions remained valid within the SHR model framework, we agreed that future studies should include WKY controls to further dissect hypertension-related mechanisms. Given that this CSVD model was induced by chronic hypertension, while our results demonstrated that NBP had no significant effect on blood pressure. This intriguing dissociation between hemodynamic and therapeutic effects warranted careful discussion. Extensive research on ischemic cerebrovascular diseases had demonstrated that NBP exerted multiple therapeutic effects including anti-inflammatory, anti-oxidative stress, protection of the BBB, and promotion of vascular regeneration effects, which were independent of its regulation of blood pressure [32–34]. This was corroborated by multiple clinical studies confirming NBP’s lack of significant hypotensive effects [35,36], which aligned perfectly with our current findings in SHR models. While this study focused on NBP’s neuroprotective mechanisms, we acknowledged that its potential effects on cerebral blood flow (CBF) dynamics in hypertensive models remained to be fully elucidated. Preliminary data from laser speckle contrast imaging suggest possible CBF improvement without systemic blood pressure changes of NBP in ischemic stroke [32,37]. To determine the potential protective mechanisms of NBP in rats with CSVD, DIA-based proteomics was applied. The most basic goal of proteomics is to comprehensively identify and quantify the entire protein and its modifications in a biological sample of interest [38,39]. DIA-based proteomics can efficiently determine low-abundance proteins in complex samples, thus significantly improving the reliability of quantitative analysis and becoming a key technology for studying cell signal transduction, drug discovery, and clinical characterization [40,41]. The results of the DIA proteomics showed that 143 proteins were upregulated and 119 proteins were downregulated significantly in NBP-treated SHR. GO analysis indicated that DEPs were involved in the regulation of immunity and inflammation. We also conducted a KEGG analysis on the differential proteins. The results showed that the FDR-adjusted *p*-value of the NF-κB pathway was greater than 0.05 (Figure S1). However, we still chose to study the NF-κB pathway in this study for three reasons as follows. First, NF-κB pathway is a well-documented pathway in hypertension-related neuroinflammation [42,43]. Second, upon receptor activation, the IKK complex is activated, facilitating NF-κB translocation into the nucleus where it binds to DNA and initiates transcription of inflammatory factors. Simultaneously, the activated IKK complex promotes phosphorylation of NF-κB, thereby modulating its transcriptional activity. Consequently, during NF-κB activation, the total cellular NF-κB protein levels may not exhibit significant changes [44]. Third, our subsequent western blot results also demonstrated that p-NF-KB was significantly downregulated after NBP intervention. TLR4 is a PRR belonging to the TLR family. Its excessive activation contributes to chronic neuroinflammation and has been implicated in the pathogenesis of various neurological disorders. TLR4 inducing the NF-κB activation pathway, is an important and classical signaling pathway involved in non-specific immunity, as well as intra-cellular inflammation [45]. When TLR4 is activated, it causes a series of signal cascades through the MyD88 dependent pathway, promotes phosphorylation and translocalization of NF-κB into the nucleus, and ultimately promotes the mass production of pro-inflammatory factors, including TNF-α and IL-1β [46]. In the SHR model, existing studies have shown that inhibition of TLR4/NF-κB signaling could ameliorate the neuroinflammation, improving the behavior of animals [14,47]. Our western blotting and PCR results showed that with the treatment of NBP, the high expression of the TLR4/MyD88/NF-κB signaling pathway and the release of inflammatory factors in the hippocampus of SHRs were decreased, which provided evidence that NBP may play a neuroprotective role by modulating the TLR4/MyD88/NF-κB pathway.

This study has several limitations. First, the sample size was relatively small, and larger sample sizes are needed to obtain more accurate results. Second, in the SHRs’ brains, with the application of NBP, the TLR4/NF-κB pathway was downregulated. This phenomenon bolstered the biological plausibility of our hypothesis that NBP may down-regulate the TLR4/MyD88/NF-κB pathway. However, this did not indicate a direct inhibitory effect of NBP on this signaling pathway. Further research should apply TLR4 inhibitors or other interventions to strengthen the results. Third, in this study, only male rats were included as the research subjects. This was because we considered that male rats were more stable and excluded the influence of estrogen. However, this was not complete enough. In future studies, female rats will definitely be included to make the research results more comprehensive and rigorous.

## 5 Conclusions

In this exploratory study, we found that NBP effectively ameliorates cognitive impairment and attenuates neuroinflammation in chronic hypertension-induced CSVD, primarily through modulation of the TLR4/NF-κB signaling pathway.

## Supplementary Material

Supplementary Figure

Supplementary Table 1

Supplementary Table 2
