# Supplementary material for: DIA-based proteomics reveals anti-inflammatory role of DL-3-n-butylphthalide in cerebral small vessel disease-induced brain injury in hypertensive rat
Source: Transl Neurosci. 2025 Aug 29;16(1):20250381. doi: 10.1515/tnsci-2025-0381 (PMC12413627; doi:10.1515/tnsci-2025-0381)
Supplement: Supplementary Figure [file tnsci-2025-0381-sm1.pdf]

# Supplementary material

Figure S1: KEGG PATHWAY enrichment analysis of DEPs in NBP- and vehicle-treated model. Table S1: the complete list of DEPs between NBP-treated CSVD rats and untreated ones. Table S2: Full DEP annotations. Table S3: the specific Z-score values of each DEPs in heatmap. Heatmap S1: the complete heatmap include each protein symbol.

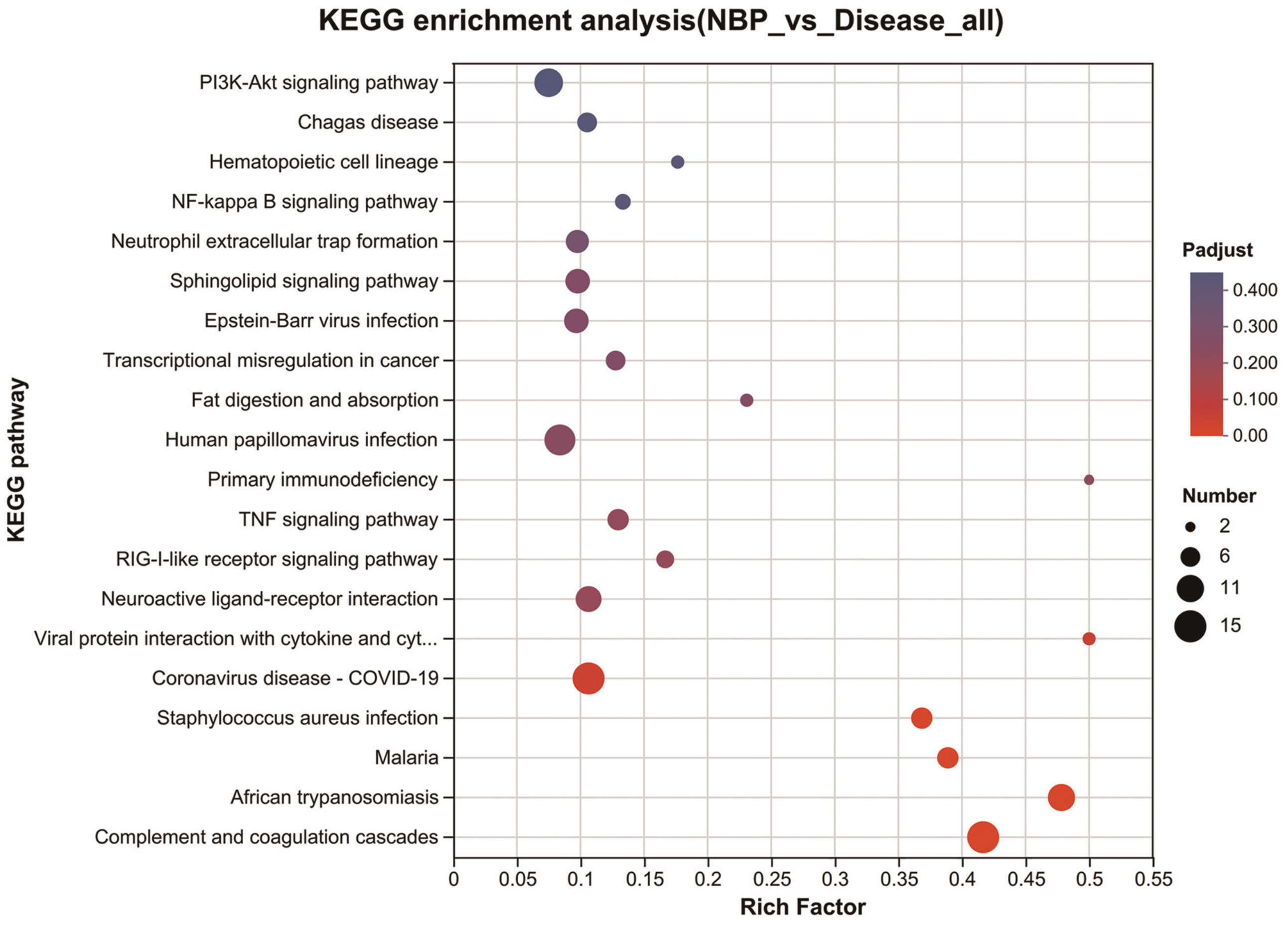


**Figure S1:** KEGG PATHWAY enrichment analysis of DEPs in NBP- and vehicle-treated model. The *X* axis is the enrichment fraction, and the *Y* axis is the KEGG pathway term. FDR-adjusted p-values was performed using the BH method to control the False Discovery Rate (FDR).

**Table S1:** DEPs between NBP group and Model one

| Accession | Protein name | Fold change | *P*_value | Regulate |
|---|---|---|---|---|
| ENSRNOP00000056637 | Slc13a4 | 0.2337 | 0.001947 | Down |
| ENSRNOP00000069564 | Neuron-specific vesicular protein calcyon | 0.3232 | 0.005498 | Down |
| ENSRNOP00000022190 | Mitogen-activated protein kinase kinase kinase 8 | 0.3459 | 0.01531 | Down |
| ENSRNOP00000031163 | H/ACA ribonucleoprotein complex non-core subunit NAF1 | 0.3536 | 0.003592 | Down |
| ENSRNOP00000062371 | Yeats2 | 0.3721 | 0.02533 | Down |
| ENSRNOP00000073891 | Taf9b | 0.374 | 0.008539 | Down |
| ENSRNOP00000020044 | Nr2f1 | 0.3952 | 0.0009046 | Down |
| ENSRNOP00000011026 | Nuclear receptor coactivator | 0.4122 | 0.0482 | Down |
| ENSRNOP00000003077 | Platelet-derived growth factor receptor alpha | 0.4456 | 0.0343 | Down |
| ENSRNOP00000017966 | Wwc2 | 0.4549 | 0.03999 | Down |
| ENSRNOP00000014772 | Tetraspanin | 0.4691 | 0.009033 | Down |
| ENSRNOP00000015283 | Transcription factor SOX-10 | 0.4757 | 0.04588 | Down |
| ENSRNOP00000087342 | Transmembrane protein 177 | 0.4977 | 0.02658 | Down |
| ENSRNOP00000026466 | Histone acetyltransferase | 0.4997 | 0.02808 | Down |
| ENSRNOP00000046751 | Unc13b | 0.5189 | 0.01348 | Down |
| ENSRNOP00000020405 | Tumor necrosis factor receptor type 1-associated DEATH domain protein | 0.5436 | 0.02098 | Down |
| ENSRNOP00000081475 | Olfactory receptor | 0.5466 | 0.01425 | Down |
| ENSRNOP00000074771 | Pid1 | 0.5483 | 0.02044 | Down |
| ENSRNOP00000027266 | Rab40c | 0.5724 | 0.01759 | Down |
| ENSRNOP00000075445 | Mitochondrial fission factor | 0.5782 | 0.004676 | Down |
| ENSRNOP00000045466 | Sulfotransferase | 0.5799 | 0.04882 | Down |
| ENSRNOP00000045475 | Atn1 | 0.5805 | 0.01104 | Down |
| ENSRNOP00000064840 | Alkaline ceramidase | 0.6041 | 0.01266 | Down |
| ENSRNOP00000053461 | Camta2 | 0.6107 | 0.01998 | Down |
| ENSRNOP00000021802 | Guanylate cyclase | 0.6261 | 0.0234 | Down |
| ENSRNOP00000014546 | Serine palmitoyltransferase 1 | 0.6327 | 0.008053 | Down |
| ENSRNOP00000008045 | Regulator of microtubule dynamics protein 2 | 0.6348 | 0.04729 | Down |
| ENSRNOP00000028375 | Glucose-6-phosphatase | 0.6351 | 0.005688 | Down |
| ENSRNOP00000072091 | Sodium channel protein | 0.6556 | 0.009328 | Down |
| ENSRNOP00000031380 | Phospholipase A2 | 0.6578 | 0.02092 | Down |
| ENSRNOP00000027528 | Asb1 | 0.6601 | 0.03794 | Down |
| ENSRNOP00000012342 | Cannabinoid receptor 2 | 0.6669 | 0.02295 | Down |
| ENSRNOP00000043928 | Paxillin | 0.6715 | 0.03345 | Down |
| ENSRNOP00000012461 | TANK-binding kinase 1-binding protein 1 | 0.6802 | 0.02159 | Down |
| ENSRNOP00000070793 | Ube2b | 0.6852 | 0.03149 | Down |
| ENSRNOP00000028288 | Elongator complex protein 6 | 0.6867 | 0.004103 | Down |
| ENSRNOP00000012942 | Plasma membrane ascorbate-dependent reductase CYBRD1 | 0.6897 | 0.00431 | Down |
| ENSRNOP00000028401 | Heat shock protein beta-6 | 0.6899 | 0.01896 | Down |
| ENSRNOP00000008760 | CD44 antigen | 0.6953 | 0.03201 | Down |
| ENSRNOP00000072503 | Unc119b | 0.6968 | 0.01533 | Down |

(*Continued*)

**Table S1:** *Continued*

| Accession | Protein name | Fold change | *P*_value | Regulate |
|---|---|---|---|---|
| ENSRNOP00000086806 | Protein-tyrosine-phosphatase | 0.6973 | 0.003621 | Down |
| ENSRNOP00000029546 | Vrk1 | 0.699 | 0.02289 | Down |
| ENSRNOP00000002465 | Sspn | 0.7106 | 0.04751 | Down |
| ENSRNOP00000001169 | Fhl1 | 0.7123 | 0.04024 | Down |
| ENSRNOP00000026646 | Chrng | 0.7237 | 0.04209 | Down |
| ENSRNOP00000017156 | Zcchc17 | 0.7417 | 0.03393 | Down |
| ENSRNOP00000044720 | Glutathione transferase | 0.7441 | 0.00626 | Down |
| ENSRNOP00000035915 | Cln6 | 0.7443 | 0.006996 | Down |
| ENSRNOP00000025546 | Protein-serine/threonine phosphatase | 0.7456 | 0.03424 | Down |
| ENSRNOP00000022958 | Signal peptidase complex catalytic subunit SEC11 | 0.7499 | 0.02387 | Down |
| ENSRNOP00000073055 | Adgra1 | 0.7522 | 0.04028 | Down |
| ENSRNOP00000003470 | Rchy1 | 0.753 | 0.002251 | Down |
| ENSRNOP00000065390 | Maleylacetoacetate isomerase | 0.756 | 0.02512 | Down |
| ENSRNOP00000072765 | Beta-mannosidase | 0.7687 | 0.03509 | Down |
| ENSRNOP00000025600 | Trim33 | 0.7689 | 0.04933 | Down |
| ENSRNOP00000020357 | SPARC-like protein 1 | 0.7718 | 0.04264 | Down |
| ENSRNOP00000078867 | Cbx1 | 0.7778 | 0.02451 | Down |
| ENSRNOP00000018003 | Hykk | 0.7801 | 0.0275 | Down |
| ENSRNOP00000092181 | Calponin-3 | 0.7838 | 0.01819 | Down |
| ENSRNOP00000019637 | Phosphodiesterase | 0.7843 | 0.002622 | Down |
| ENSRNOP00000026499 | Mrps18a | 0.7844 | 0.01258 | Down |
| ENSRNOP00000018877 | Il6st | 0.7854 | 0.03672 | Down |
| ENSRNOP00000075305 | Soga1 | 0.7871 | 0.02141 | Down |
| ENSRNOP00000059566 | EEF1A lysine methyltransferase 1 | 0.7945 | 0.01272 | Down |
| ENSRNOP00000026249 | Craniofacial development protein 1 | 0.7952 | 0.01647 | Down |
| ENSRNOP00000004121 | ADP-ribosyl cyclase/cyclic ADP-ribose hydrolase 1 | 0.7953 | 0.006096 | Down |
| ENSRNOP00000016464 | Exosc4 | 0.7994 | 0.03956 | Down |
| ENSRNOP00000071115 | Netrin receptor UNC5 | 0.8025 | 0.04591 | Down |
| ENSRNOP00000044914 | Protein OS-9 | 0.8039 | 0.02854 | Down |
| ENSRNOP00000093820 | TIP41-like protein | 0.8044 | 0.03105 | Down |
| ENSRNOP00000064843 | L1 transposable element | 0.8044 | 0.006844 | Down |
| ENSRNOP00000028328 | Bax | 0.8107 | 0.01975 | Down |
| ENSRNOP00000018487 | Ighmbp2 | 0.8119 | 0.01994 | Down |
| ENSRNOP00000002579 | Ranbp1 | 0.812 | 0.03199 | Down |
| ENSRNOP00000081021 | Irf2bp2 | 0.8182 | 0.02133 | Down |
| ENSRNOP00000013563 | Sdcbp | 0.8185 | 0.02965 | Down |
| ENSRNOP00000015756 | Rpl22l1 | 0.8218 | 0.02839 | Down |
| ENSRNOP00000071072 | Phosphatidylserine synthase | 0.8221 | 0.03044 | Down |
| ENSRNOP00000024113 | Ercc1 | 0.8252 | 0.03601 | Down |
| ENSRNOP00000045007 | 39S ribosomal protein L17, mitochondrial | 0.826 | 0.04778 | Down |

(*Continued*)

**Table S1:** *Continued*

| Accession | Protein name | Fold change | *P*_value | Regulate |
|---|---|---|---|---|
| ENSRNOP00000025512 | C19h1orf198 | 0.8287 | 0.03133 | Down |
| ENSRNOP00000047508 | Alpha/beta hydrolase domain-containing protein 17B | 0.8292 | 0.034 | Down |
| ENSRNOP00000093020 | Fam98c | 0.8293 | 0.04759 | Down |
| ENSRNOP00000072616 | Ap5s1 | $1.0 \times 10^{-5}$ | 0.001216 | Down |
| ENSRNOP00000048288 | Solute carrier organic anion transporter family member | $1.0 \times 10^{-5}$ | 0.003882 | Down |
| ENSRNOP00000052126 | Cep250 | $1.0 \times 10^{-5}$ | 0.04694 | Down |
| ENSRNOP00000022552 | Phyhd1 | $1.0 \times 10^{-5}$ | 0.02217 | Down |
| ENSRNOP00000035420 | Crb2 | $1.0 \times 10^{-5}$ | 0.0041 | Down |
| ENSRNOP00000069668 | Tmem63a | $1.0 \times 10^{-5}$ | 0.009543 | Down |
| ENSRNOP00000083031 | Choline transporter-like protein | $1.0 \times 10^{-5}$ | 0.004217 | Down |
| ENSRNOP00000052057 | Tmub2 | $1.0 \times 10^{-5}$ | 0.003392 | Down |
| ENSRNOP00000084023 | Nucleolar protein 6 | $1.0 \times 10^{-5}$ | 0.01434 | Down |
| ENSRNOP00000012883 | Squalene monooxygenase | $1.0 \times 10^{-5}$ | 0.005037 | Down |
| ENSRNOP00000006365 | Putative tRNA (cytidine(32)/guanosine(34)-2'-O)-methyltransferase | $1.0 \times 10^{-5}$ | 0.01768 | Down |
| ENSRNOP00000090773 | Lrsam1 | $1.0 \times 10^{-5}$ | 0.009332 | Down |
| ENSRNOP00000006519 | Llgl2 | $1.0 \times 10^{-5}$ | 0.02671 | Down |
| ENSRNOP00000020925 | Fbxo4 | $1.0 \times 10^{-5}$ | 0.04486 | Down |
| ENSRNOP00000023099 | Trim5 | $1.0 \times 10^{-5}$ | 0.008198 | Down |
| ENSRNOP00000080123 | Mettl27 | $1.0 \times 10^{-5}$ | 0.01133 | Down |
| ENSRNOP00000080269 | Kcnj16 | $1.0 \times 10^{-5}$ | 0.03101 | Down |
| ENSRNOP00000038464 | Pecanex-like protein | $1.0 \times 10^{-5}$ | 0.02542 | Down |
| ENSRNOP00000040411 | Receptor protein-tyrosine kinase | $1.0 \times 10^{-5}$ | 0.01616 | Down |
| ENSRNOP00000069304 | Zfand3 | $1.0 \times 10^{-5}$ | 0.03561 | Down |
| ENSRNOP00000046455 | Mitogen-activated protein kinase 12 | $1.0 \times 10^{-5}$ | 0.002546 | Down |
| ENSRNOP00000077569 | Nat8f5 | $1.0 \times 10^{-5}$ | 0.01442 | Down |
| ENSRNOP00000011115 | Crocc | $1.0 \times 10^{-5}$ | 0.005209 | Down |
| ENSRNOP00000043075 | Sirpb2 | $1.0 \times 10^{-5}$ | 0.01156 | Down |
| ENSRNOP00000044081 | Hba | $1.0 \times 10^{-5}$ | 0.01942 | Down |
| ENSRNOP00000034774 | AABR07065265 | $1.0 \times 10^{-5}$ | 0.01394 | Down |
| ENSRNOP00000016265 | Snx10 | $1.0 \times 10^{-5}$ | 0.03464 | Down |
| ENSRNOP00000001322 | Stxbp2 | $1.0 \times 10^{-5}$ | 0.02178 | Down |
| ENSRNOP00000024655 | Dhx32 | $1.0 \times 10^{-5}$ | 0.04386 | Down |
| ENSRNOP00000088808 | Pcdhga5 | $1.0 \times 10^{-5}$ | 0.02344 | Down |
| ENSRNOP00000030489 | Cfap298 | $1.0 \times 10^{-5}$ | 0.01689 | Down |
| ENSRNOP00000003343 | Ifi47 | $1.0 \times 10^{-5}$ | 0.04058 | Down |
| ENSRNOP00000067032 | Suds3 | $1.0 \times 10^{-5}$ | 0.007732 | Down |
| ENSRNOP00000011663 | PDZ and LIM domain protein 2 | $1.0 \times 10^{-5}$ | 0.01034 | Down |
| ENSRNOP00000000529 | Tap1 | $1.0 \times 10^{-5}$ | 0.04995 | Down |
| ENSRNOP00000054248 | Tgfa | $1.0 \times 10^{-5}$ | 0.03702 | Down |
| ENSRNOP00000063969 | Decreased expression in renal and prostate cancer protein | 1.2 | 0.03076 | Up |

(*Continued*)

**Table S1:** *Continued*

| Accession | Protein name | Fold change | *P*_value | Regulate |
|---|---|---|---|---|
| ENSRNOP00000021839 | Tmem67 | 1.2 | 0.03154 | Up |
| ENSRNOP00000011556 | Wdr5 | 1.202 | 0.03458 | Up |
| ENSRNOP00000073719 | KH type-2 domain-containing protein | 1.203 | 0.02987 | Up |
| ENSRNOP00000061062 | Receptor expression-enhancing protein | 1.209 | 0.01961 | Up |
| ENSRNOP00000021381 | Tetraspanin-5 | 1.215 | 0.0382 | Up |
| ENSRNOP00000006768 | Eukaryotic translation initiation factor 2D | 1.215 | 0.04208 | Up |
| ENSRNOP00000074749 | THO complex subunit 2 | 1.217 | 0.02423 | Up |
| ENSRNOP00000020663 | Serine/threonine-protein phosphatase 2A catalytic subunit beta isoform | 1.221 | 0.04166 | Up |
| ENSRNOP00000080482 | Dlgap2 | 1.239 | 0.03624 | Up |
| ENSRNOP00000026680 | Ankrd28 | 1.239 | 0.005147 | Up |
| ENSRNOP00000049726 | Echdc2 | 1.262 | 0.02649 | Up |
| ENSRNOP00000068751 | Peroxin-13 | 1.283 | 0.01423 | Up |
| ENSRNOP00000018888 | Ubiquitin carboxyl-terminal hydrolase CYLD | 1.289 | 0.04871 | Up |
| ENSRNOP00000026962 | Folr1 | 1.296 | 0.03972 | Up |
| ENSRNOP00000071841 | Prpf40b | 1.297 | 0.0436 | Up |
| ENSRNOP00000084499 | B3glct | 1.298 | 0.03656 | Up |
| ENSRNOP00000065376 | 39S ribosomal protein L40, mitochondrial | 1.3 | 0.02778 | Up |
| ENSRNOP00000097555 | COX assembly mitochondrial protein | 1.302 | 0.02797 | Up |
| ENSRNOP00000072186 | DNA-directed RNA polymerase II subunit GRINL1A | 1.306 | 0.01751 | Up |
| ENSRNOP00000066790 | NADH dehydrogenase [ubiquinone] 1 alpha subcomplex subunit 3 | 1.309 | 0.04163 | Up |
| ENSRNOP00000066577 | Glutathione peroxidase | 1.315 | 0.035 | Up |
| ENSRNOP00000022578 | Transmembrane protein 135 | 1.326 | 0.04968 | Up |
| ENSRNOP00000022900 | NAD(+) kinase | 1.332 | 0.04998 | Up |
| ENSRNOP00000018032 | Sharpin | 1.333 | 0.03252 | Up |
| ENSRNOP00000076969 | Ociad2 | 1.345 | 0.03079 | Up |
| ENSRNOP00000077626 | Nudt4 | 1.35 | 0.03739 | Up |
| ENSRNOP00000003021 | Polyprenol reductase | 1.352 | 0.02257 | Up |
| ENSRNOP00000075738 | Protein arginine *N*-methyltransferase | 1.388 | 0.03154 | Up |
| ENSRNOP00000004717 | Sodium-dependent serotonin transporter | 1.389 | 0.02431 | Up |
| ENSRNOP00000028912 | Dusp23 | 1.402 | 0.03376 | Up |
| ENSRNOP00000055288 | 40S ribosomal protein S2 | 1.421 | 0.03465 | Up |
| ENSRNOP00000092022 | Gamma-tubulin complex component | 1.422 | 0.03695 | Up |
| ENSRNOP00000082905 | Dst | 1.425 | 0.01553 | Up |
| ENSRNOP00000048165 | KRAB domain-containing protein | 1.435 | 0.0005937 | Up |
| ENSRNOP00000010238 | Lrtm2 | 1.448 | 0.03349 | Up |
| ENSRNOP00000024330 | Phosphatidylinositol-3,4-bisphosphate 4-phosphatase | 1.455 | 0.001831 | Up |
| ENSRNOP00000016582 | Ribosomal RNA-processing protein 40 | 1.474 | 0.03299 | Up |
| ENSRNOP00000020770 | Lon protease homolog 2, peroxisomal | 1.513 | 0.03556 | Up |
| ENSRNOP00000058007 | Itih4 | 1.519 | 0.04615 | Up |
| ENSRNOP00000020065 | MAP kinase-activated protein kinase 3 | 1.523 | 0.03227 | Up |

(*Continued*)

**Table S1:** *Continued*

| Accession | Protein name | Fold change | *P*_value | Regulate |
|---|---|---|---|---|
| ENSRNOP00000019117 | Prepronociceptin | 1.545 | 0.005356 | Up |
| ENSRNOP00000008168 | WD repeat, SAM and U-box domain-containing protein 1 | 1.546 | 0.02707 | Up |
| ENSRNOP00000012725 | Signal recognition particle receptor subunit beta | 1.572 | 0.04987 | Up |
| ENSRNOP00000090709 | Isg15 | 1.576 | 0.04668 | Up |
| ENSRNOP00000081883 | Chn1 | 1.618 | 0.04968 | Up |
| ENSRNOP00000023140 | Ataxin-1 | 1.621 | 0.03313 | Up |
| ENSRNOP00000033462 | Rrp7a | 1.622 | 0.03724 | Up |
| ENSRNOP00000012495 | Presenilin-1 | 1.632 | 0.01522 | Up |
| ENSRNOP00000014912 | Translocon-associated protein subunit gamma | 1.639 | 0.004824 | Up |
| ENSRNOP00000059783 | Integrin beta | 1.639 | 0.01113 | Up |
| ENSRNOP00000061606 | Dysf | 1.643 | 0.04995 | Up |
| ENSRNOP00000081181 | Strip2 | 1.685 | 0.04595 | Up |
| ENSRNOP00000056859 | Cfh | 1.69 | 0.0199 | Up |
| ENSRNOP00000069491 | Eeig2 | 1.727 | 0.02926 | Up |
| ENSRNOP00000042417 | Lrch3 | 1.745 | 0.00252 | Up |
| ENSRNOP00000019172 | Zinc transporter 7 | 1.809 | 0.03181 | Up |
| ENSRNOP00000002486 | Transmembrane 7 superfamily member 3 | 1.809 | 0.005958 | Up |
| ENSRNOP00000018961 | High affinity nerve growth factor receptor | 1.843 | 0.02581 | Up |
| ENSRNOP00000047801 | Lrch1 | 1.861 | 0.03931 | Up |
| ENSRNOP00000013476 | Protein UXT | 1.876 | 0.002088 | Up |
| ENSRNOP00000019304 | P2Y purinoceptor 1 | 1.878 | 0.02007 | Up |
| ENSRNOP00000007919 | Slc30a6 | 1.948 | 0.004996 | Up |
| ENSRNOP00000017883 | Rbpms | 1.969 | 0.006784 | Up |
| ENSRNOP00000087322 | Gap junction gamma-1 protein | 2.132 | 0.01066 | Up |
| ENSRNOP00000028793 | Thioredoxin-interacting protein | 2.149 | 0.02124 | Up |
| ENSRNOP00000038525 | Retinoic acid receptor responder protein 2 | 2.157 | 0.04137 | Up |
| ENSRNOP00000009503 | Serbp1 | 2.158 | 0.004766 | Up |
| ENSRNOP00000087389 | Non-specific serine/threonine protein kinase | 2.36 | 0.03529 | Up |
| ENSRNOP00000070342 | Glutathione peroxidase | 2.397 | 0.000729 | Up |
| ENSRNOP00000068681 | Myo15a | 2.463 | 0.03572 | Up |
| ENSRNOP00000055275 | ADP/ATP translocase | 2.52 | 0.01971 | Up |
| ENSRNOP00000015687 | G protein-coupled receptor kinase | 2.571 | 0.03394 | Up |
| ENSRNOP00000079912 | Gp_dh_C domain-containing protein | 2.608 | 0.03855 | Up |
| ENSRNOP00000007832 | Ttc21b | 2.627 | 0.001781 | Up |
| ENSRNOP00000079541 | Ubiquitin-60S ribosomal protein L40 | 2.641 | 0.0002397 | Up |
| ENSRNOP00000045637 | Serotransferrin | 2.648 | 0.01532 | Up |
| ENSRNOP00000026643 | von Willebrand factor | 2.818 | 0.01233 | Up |
| ENSRNOP00000046622 | Fibulin-1 | 2.898 | 0.0134 | Up |
| ENSRNOP00000001759 | Adenosine receptor A2a | 2.977 | 0.03888 | Up |
| ENSRNOP00000011983 | Slc18a2 | 3.19 | 0.01162 | Up |

(*Continued*)

**Table S1:** *Continued*

| Accession | Protein name | Fold change | *P*_value | Regulate |
|---|---|---|---|---|
| ENSRNOP00000004716 | Spta1 | 3.254 | 0.01705 | Up |
| ENSRNOP00000014661 | Tox | 3.312 | 0.02047 | Up |
| ENSRNOP00000000611 | Colipase | 3.423 | 0.04715 | Up |
| ENSRNOP00000069937 | Hbb | 4.004 | 0.01866 | Up |
| ENSRNOP00000022012 | Gtse1 | 4.053 | 0.03114 | Up |
| ENSRNOP00000029319 | Ig kappa chain V region | 4.091 | 0.04733 | Up |
| ENSRNOP00000077625 | LOC297568 | 4.786 | 0.03931 | Up |
| ENSRNOP00000051504 | Fetub | 4.877 | 0.03532 | Up |
| ENSRNOP00000075693 | Serpina3a | 4.959 | 0.001046 | Up |
| ENSRNOP00000066934 | Complement C3 | 5.15 | 0.02996 | Up |
| ENSRNOP00000071685 | Cfi | 5.156 | 0.0003157 | Up |
| ENSRNOP00000091885 | Hbb | 5.43 | 0.03889 | Up |
| ENSRNOP00000012577 | Serpina1 | 5.498 | 0.02419 | Up |
| ENSRNOP00000081763 | GLOBIN domain-containing protein | 5.634 | 0.03757 | Up |
| ENSRNOP00000086253 | Kng2 | 5.96 | 0.04802 | Up |
| ENSRNOP00000065206 | Apolipoprotein A-I | 6.055 | 0.04564 | Up |
| ENSRNOP00000041451 | Hba | 6.665 | 0.03093 | Up |
| ENSRNOP00000068726 | Hbb | 6.912 | 0.02761 | Up |
| ENSRNOP00000068875 | Hbb | 6.988 | 0.02271 | Up |
| ENSRNOP00000040010 | Hbe1 | 7.606 | 0.02551 | Up |
| ENSRNOP00000014267 | Carbonic anhydrase 1 | 7.66 | 0.03031 | Up |
| ENSRNOP00000043166 | Gp_dh_N domain-containing protein | 8.163 | 0.0003314 | Up |
| ENSRNOP00000074568 | Anion exchange protein | 8.332 | 0.01598 | Up |
| ENSRNOP00000060007 | Fibrinogen alpha chain | 9.607 | 0.01346 | Up |
| ENSRNOP00000004174 | Vitamin D-binding protein | 9.918 | 0.01137 | Up |
| ENSRNOP00000024710 | Hemopexin | 10.15 | 0.0007413 | Up |
| ENSRNOP00000009813 | Fibrinogen beta chain | 13.22 | 0.00524 | Up |
| ENSRNOP00000032735 | Fibrinogen gamma chain | 15.31 | 0.01218 | Up |
| ENSRNOP00000021531 | Endonuclease | 15.47 | 0.01452 | Up |
| ENSRNOP00000020196 | Haptoglobin | 16.8 | 0.01133 | Up |
| ENSRNOP00000075573 | Kng2 | 20.14 | 0.003753 | Up |
| ENSRNOP00000075264 | Kng1 | 22.98 | 0.01578 | Up |
| ENSRNOP00000010454 | Orm1 | 45.96 | 0.04931 | Up |
| ENSRNOP00000023282 | Adad1 | 45.96 | 0.00298 | Up |
| ENSRNOP00000010783 | Trimethylguanosine synthase | 45.96 | 0.009264 | Up |
| ENSRNOP00000089235 | Ccdc146 | 45.96 | 0.001125 | Up |
| ENSRNOP00000016897 | Galectin | 45.96 | 0.0133 | Up |
| ENSRNOP00000022713 | Itga8 | 45.96 | 0.00532 | Up |
| ENSRNOP00000034820 | D(1A) dopamine receptor | 45.96 | 0.01288 | Up |
| ENSRNOP00000006533 | Cyp4f1 | 45.96 | 0.02684 | Up |

(*Continued*)

**Table S1:** *Continued*

| Accession | Protein name | Fold change | *P*_value | Regulate |
|---|---|---|---|---|
| ENSRNOP00000084506 | Sgsh | 45.96 | 0.001974 | Up |
| ENSRNOP00000041658 | Ig-like domain-containing protein | 45.96 | 0.01376 | Up |
| ENSRNOP00000015351 | S100a9 | 45.96 | 0.02562 | Up |
| ENSRNOP00000019014 | B-cell linker protein | 45.96 | 0.04056 | Up |
| ENSRNOP00000018244 | Forkhead box protein O1 | 45.96 | 0.01979 | Up |
| ENSRNOP00000039704 | Nhs | 45.96 | 0.005135 | Up |
| ENSRNOP00000062325 | Steroid 11-beta-monooxygenase | 45.96 | 0.01355 | Up |
| ENSRNOP00000012881 | Ccdc120 | 45.96 | 0.01208 | Up |
| ENSRNOP00000086018 | Ig-like domain-containing protein | 45.96 | 0.01297 | Up |
| ENSRNOP00000026335 | Mediator of RNA polymerase II transcription subunit 11 | 45.96 | 0.01152 | Up |
| ENSRNOP00000035575 | Chromatin-remodeling ATPase INO80 | 45.96 | 0.01677 | Up |
| ENSRNOP00000059436 | Ig-like domain-containing protein | 45.96 | 0.005397 | Up |
| ENSRNOP00000077941 | Gm6569 | 45.96 | 0.003586 | Up |
| ENSRNOP00000021362 | Engulfment and cell motility protein 3 | 45.96 | 0.04716 | Up |
| ENSRNOP00000018162 | Cysteine-rich secretory protein 1 | 45.96 | 0.01453 | Up |
| ENSRNOP00000063855 | RING finger protein 10 | 45.96 | 0.008065 | Up |
| ENSRNOP00000040918 | Spata19 | 45.96 | 0.02034 | Up |
| ENSRNOP00000027512 | Pdcd11 | 45.96 | 0.04021 | Up |
| ENSRNOP00000011823 | Paraoxonase | 45.96 | 0.03821 | Up |
| ENSRNOP00000033812 | Poly [ADP-ribose] polymerase | 45.96 | 0.03748 | Up |
| ENSRNOP00000015190 | S-adenosylmethionine synthase | 45.96 | 0.01794 | Up |
| ENSRNOP00000025966 | Npr3 | 45.96 | 0.03248 | up |

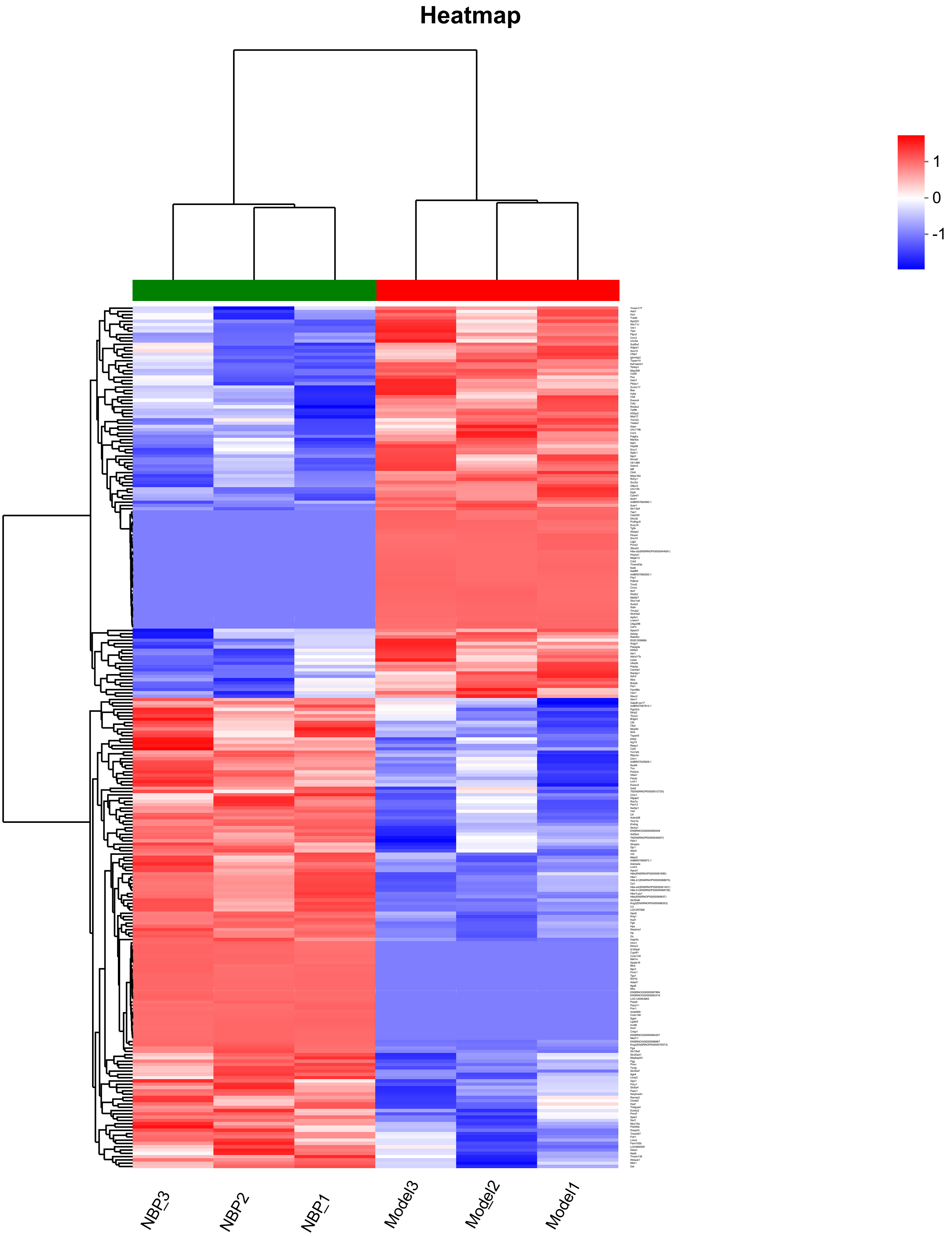
Heatmap
1
0
-1
NBP3
NBP2
NBP1
Model3
Model2
Model1
